# Mechanisms of resistance to PARP inhibitors - an evolving challenge in oncology

**DOI:** 10.20517/cdr.2019.50

**Published:** 2019-09-19

**Authors:** Angela Mweempwa, Michelle K. Wilson

**Affiliations:** Cancer and Blood, Auckland City Hospital, Auckland 1023, New Zealand.

**Keywords:** Poly-adenosine diphosphate ribose polymerase inhibition, resistance mechanisms, homologous recombination, BRCA reversion, ovarian cancer

## Abstract

Poly-adenosine diphosphate ribose polymerase inhibitors (PARPi) lead to synthetic lethality when used in cancers harbouring a BRCA mutation or homologous recombination deficiency. There are now four PARPi approved by the Food and Drug Administration for therapeutic use is ovarian and breast cancer. In addition to this, there is data supporting its use in pancreatic adenocarcinoma and prostate cancer. However, development of resistance to PARPi limits the duration of response. Key mechanisms found to date include: (1) restoration of homologous recombination; (2) changes in PARP1; (3) suppression of non-homologous end joining; (4) replication fork protection; and (5) drug concentration. Gaining a better understanding of resistance mechanisms may guide combination therapies to overcome the resistance and improve the efficacy of PARPi. The purpose of this review is to describe the resistance mechanisms to PARPi and discuss their early detection.

## Introduction

Poly-adenosine diphosphate (ADP) ribose polymerase (PARP) inhibitors are now part of mainstream therapy for cancers harbouring BRCA mutations as well as other deficiencies in homologous recombination (HR) repair^[1-6]^. Since 2014, four PARP inhibitors (PARPi) have been approved in the United States: olaparib, niraparib, rucaparib and talazoparib^[3,5,7-12]^ [Table 1]. Olaparib was the first FDA approved PARPi, initially for recurrent ovarian carcinoma with a germline BRCA mutation exposed to at least 3 lines of therapy and subsequently as maintenance therapy for women with germline BRCA mutations and patients with breast cancer with germline BRCA mutations^[1,2,5,7-9]^. Veliparib was awarded orphan drug designation for squamous non-small cell lung cancer^[13]^. Although veliparib is the least potent of the PARPi currently in use, there are multiple combination trials in progress such as the S1416 study (NCT02595905)^[14]^. Pamiparib is another PARPi under investigation in a number of clinical trials^[15-17]^.

**Table 1:** Current FDA approved PARP inhibitors

Drug	FDA approval	Indications	mPFS (hazard ratio, *P*-value)	mOS (hazard ratio, *P*-value)	Selected characteristics
Olaparib	2014	Recurrent ovarian cancer with a germline BRCA mutation after 3 lines of therapy^[9]^	6.7 months	Not reported	Creatinine elevation in 11% of patients (any grade)^[5]^
	2017	Maintenance therapy for recurrent ovarian carcinoma in CR or PR following platinum-based therapy^[5]^	19.1 months (hazard ratio 0.30, *P* < 0.0001)	Immature	
	2018	Chemotherapy exposed HER2 negative breast cancer with known or suspected germline BRCA mutation^[7]^	7 months (hazard ratio 0.58, *P* < 0.001)	19.3 months (hazard ratio 0.90, *P* = 0.57)	
	2018	First line maintenance in advanced ovarian cancer with germline BRCA mutations in CR or PR after platinum-based chemotherapy^[8]^	Not reached (hazard ratio 0.30, *P* < 0.001)	Not reported	
Niraparib	2017	Maintenance therapy in ovarian cancer following first line platinum chemotherapy in CR or PR^[3]^	Germline BRCAm 21 months (hazard ratio 0.27, *P* < 0.001) Non-germline BRCAm/HRD+ 12.9 months (hazard ratio 0.38, *P* < 0.001) Non-germline BRCAm 9.3 months (hazard ratio 0.45, *P* < 0.001)	Immature	Rate of grade 3 and 4 thrombocytopenia is > 30% and neutropenia 20%^[3]^ Transaminase elevation in a 1/3 of patients
Rucaparib	2016	Germline BRCAm after 2 or more lines of therapy^[10]^	10 months (integrated population)	Not reported	Transaminase elevation in a 1/3 of patients (any grade)^[10]^
	2018	Maintenance therapy in recurrent ovarian cancer following response to platinum chemotherapy^[11]^	BRCAm 16.6 months (hazard ratio 0.23, *P* < 0.0001) HRD+ 13.6 months (hazard ratio 0.32, *P* < 0.0001)	Immature	
Talazoparib	2018	Germline BRCA mutation or suspected germline BRCA mutation in HER2 negative locally advanced or metastatic breast cancer^[12]^	8.6 months (hazard ratio 0.54, *P* < 0.001)	22.3 months (hazard ratio 0.76, *P* = 0.11)	Highest potency and PARP-trapping ability^[14,18]^

FDA: Food and Drug Administration; mPFS: median progression free survival; mOS: median overall survival; CR: complete response; PR: partial response; BRCAm: BRCA mutation; HRD: homologous recombination deficiency

Talazoparib has the greatest PARP trapping activity (rank from highest: talazoparib > niraparib > olaparib = rucaparib > veliparib) and is the most potent among the PARPi in current use^[14,18]^.

Although nausea, fatigue and anaemia are common to all PARPi, some differences in the toxicity profiles have been observed. Thrombocytopenia is more prominent with niraparib, with grade 3 or 4 reductions in platelet counts occurring in a third of treated patients^[3]^. A retrospective analysis has shown that modifying the dose of niraparib based on baseline body weight and platelet counts may reduce the degree of thrombocytopenia^[19]^. Elevated serum creatinine has been observed with olaparib in 11% of patients whereas both rucaparib and niraparib have been reported to cause an increase in alanine aminotransferase or aspartate aminotransferase^[5,10]^.

The interest in these agents has expanded and is now being explored in many different cancer types including prostate and pancreatic cancer^[20,21]^. Because the likelihood of relapse is high in these patient populations, defining the mechanisms promoting resistance, and subsequent therapeutic failure remains extremely important. This paper will present the current literature on this.

## Mechanism of action of PARPi

PARPs are a superfamily of 17 enzymes involved in a multitude of intracellular processes including DNA damage repair, replication and transcription. PARP1 and PARP2 are constitutively expressed but only become activated on binding to sites of DNA damage. PARP1 and PARP2 use nicotinamide adenine dinucleotide (NAD+) as a substrate to transfer ADP-ribosyl groups onto acceptor proteins and produce long poly(ADP-ribose) (PAR) chains in a process called PARylation^[22]^. PARylation of core histones exerts a negative charge and leads to relaxation of chromatin and recruitment of transcription proteins^[23]^. Auto-PARylation of PARP1 and PARP2 permits their dissociation from DNA, a step required for the repair proteins to access damaged DNA^[24,25]^. PARylation is counteracted by the activity of PAR glycohydrolase (PARG)^[26]^.

**Figure 1:**
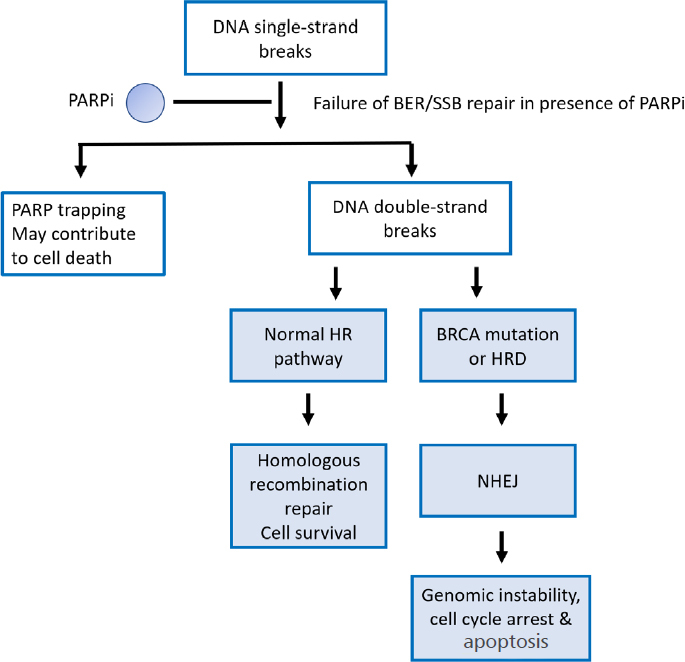
Synthetic lethality. There is failure of DNA base excision/single-strand break repair in the presence of PARPi. This causes stalling of the replication fork and subsequently collapse, leading to double-strand breaks which are repaired by homologous recombination when this pathway is intact. In the presence of a BRCA mutation or HRD, DNA double-strand breaks are repaired by NHEJ leading to genomic instability, cell cycle arrest and apoptosis. PARP inhibition in the presence of a BRCA mutation is synthetically lethal. PARP: poly-adenosine diphosphate ribose polymerase; PARPi: poly-adenosine diphosphate ribose polymerase inhibitors; BER: base excision repair; SSB: single-strand break; NHEJ: non-homologous end joining; HRD: homologous recombination deficiency; DNA: deoxyribonucleic acid

PARP1, the most abundant of the PARPs, recognizes DNA single-strand breaks and repairs them by base excision repair (BER). PARPi suppress BER leading to stalling of the replication fork and subsequent development of double-strand breaks (DSBs). In BRCA proficient cells, DSBs are repaired by HR. HR is a DNA repair mechanism that utilises a homologous chromosome or sister chromatid as a template for repair of the damaged portion of DNA^[24]^. During HR, BRCA1 promotes the 5’ to 3’ resection of DSB with the involvement of nucleases such as MRE11, carboxy-terminal binding protein interacting protein (CtIP) and exonuclease 1, leaving a 3’ overhang onto which BRCA2 loads RAD51, enabling invasion of a sister chromatid to be used as a template for DNA repair^[27]^. As a result, this repair pathway is precise and error-free^[24]^. In contrast, DSBs in BRCA deficient cells are repaired by non-homologous end joining (NHEJ), a process inherently prone to errors as it involves removal of the section containing damaged DNA and ligation of the ends which leads to loss of DNA sequence^[24]^. Errors resulting from NHEJ lead to genomic instability, cell cycle arrest and apoptosis^[28]^. It is widely known that inhibiting PARP in the setting of a BRCA mutation leads to synthetic lethality since it is the combination of a PARPi and BRCA deficiency that induces lethality^[29]^ [Figure 1].

Two mechanisms of action of PARPi have been described. PARPi competitively bind to the NAD+ site of PARP1 and PARP2 resulting in inhibition of their catalytic activity^[14,30]^. When this occurs there is a failure of auto-PARylation of PARP leading to PARP trapping, which may be the main mechanism of action of some PARPi such as talazoparib^[31]^. Although the efficacy of PARPi is higher in cancers with BRCA mutations or HR deficiency (HRD), it is known that they can be beneficial to patients with wild-type BRCA and in the absence of HRD^[3]^.

## Resistance mechanisms

**Figure 2:**
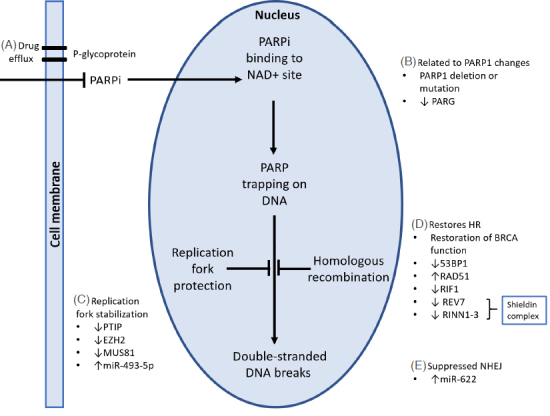
Mechanisms of PARPi resistance. A: Efflux of PARP inhibitors by P-gp pumps may contribute to resistance by reducing the intracellular PARPi concentration; B: PARP1 deletion or point mutations in PARP1 reduce sensitivity to PARPi. PARG suppression restores downstream PARP1 signalling with PARPi therapy; C: loss of expression of PTIP, EZH2, MUS81 or increased miR-493-5p results in stabilization of the replication fork, leading to PARPi resistance; D: deficiency of 53BP1, RIF1, REV7 and RINN1-3 involved in the regulation of DNA end-resection during DNA repair may induce PARPi resistance; E: overexpression of miR-622 suppresses NHEJ and rescues the homologous recombination deficiency of BRCA mutated cells. Modified from Thomas *et al.*^[30]^. PARP1: poly(ADP) ribose polymerase 1; NAD: nicotinamide adenine dinucleotide; PARG: poly(ADP) ribose glycohydrolase; HR: homologous recombination; NHEJ: non-homologous end joining; DNA: deoxyribonucleic acid

While BRCA 1/2 mutations remain the strongest markers of PARPi sensitivity^[3]^, 40%-70% of this patient cohort will fail to respond^[32-34]^. To date, several mechanisms of resistance have been described including :(1) restoration of HR; (2) changes in PARP1; (3) suppression of NHEJ; (4) replication fork protection; and (5) drug concentration [Figure 2]. The most common acquired mechanism of resistance appears to be the restoration of BRCA1 or BRCA2 protein function as a result of secondary mutations^[28]^.

## Mechanisms of resistance related to restoration of homologous recombination

There are multiple different pathways that lead to the restoration of HR function. BRCA1 or BRCA2 protein functionality can be restored through the development of intragenic mutations or epigenetic reversions that lead to activation of the open reading frame^[27]^. It is recognised that 46% of recurrent platinum-resistant epithelial ovarian carcinoma develop secondary somatic mutations restoring BRCA1/2 function^[35]^. Additionally, overexpression of BRCA driven by copy number gain and/or upregulation of the remaining functional allele has been shown in tumours with somatic loss of BRCA^[36]^.

Furthermore, *de novo* intrachromosomal genomic BRCA1 rearrangements and promoter demethylation leading to re-expression of BRCA1 have been demonstrated in PDX models and post-treatment biopsies of triple-negative breast cancer patients, respectively^[37]^. Promoter demethylation may also arise from a heterogeneous tumour in which cells with less promoter methylation undergo positive selection with exposure to PARPi^[27]^.

Additionally, other proteins involved in the DNA repair process may restore HR. Illustrating this, 53BP1 works in close relation with BRCA1 balancing HR and NHEJ repair pathways. 53BP1 commits DNA repair to classical-NHEJ by blocking CtIP-mediated DNA end resection^[27]^. The loss of 53BP1 in BRCA deficient cells leads to resection of DNA double-stranded breaks prompting RAD51 recruitment and restoration of HR^[38]^. More recently, new DNA repair factors, including RINN1, RINN2 and RINN3, have been identified which interact with REV7 to form the shieldin complex^[39-41]^. It has been proposed that shieldin functions as a downstream effector of 53BP1-RIF1 in the DNA double-stand break repair pathway, preventing DNA end resection and thus, promoting NHEJ^[39]^. Deletion of any components of the shieldin complex confers resistance to PARPi in BRCA1-mutant cells by re-establishing end resection and restoring HR^[28,39]^.

RAD51 is another protein that plays a key role in HR repair. It is loaded onto single-stranded and double-stranded DNA by BRCA2 to form a protective filament against nucleases^[42]^. An increase in RAD51 will promote HR and subsequently, resistance to PARPi^[43]^. RAD51C and RAD51D are RAD51 paralogs that also play an essential role in DNA repair through HR. The presence of a germline mutation in RAD51C and RAD51D predisposes women to ovarian cancer and is associated with PARPi sensitivity^[44]^. Secondary mutations in RAD51C and RAD51D lead to the restoration of the open reading frame, allowing HR function to proceed which contributes to the development of PARPi resistance^[44]^. PTEN loss in the setting of BRCA1 mutation is another mechanism that leads to reversal of HR deficiency^[45]^.

Acute myeloid leukaemia driven by transcription factors, AML1-ETO and PML-RARα fusion oncoproteins, is sensitive to PARPi due to suppressed HR and compromised DNA damage response^[46]^. It has been shown that overexpression of the myeloid leukaemia-associated HOXA9 gene in AML1-ETO and PML–RARα-transformed cells enhances HR efficiency leading to PARPi resistance^[46]^.

Schlafen 11 (SLFN11) expression is another factor that has been shown to correlate with PARPi sensitivity^[47,48]^. It has been suggested that SLFN11 inhibits replication by inducing prolonged cell cycle arrest at S-phase after PARPi treatment, whereas SLFN11-deficient cells are able to continue replicating, resulting in PARPi resistance^[47]^. It is likely over the next 5 years, more genes involved in this process will become recognised as evidence arises.

## Resistance mechanisms related to PARP1

PARP1 is the most abundant of the PARPs but while it plays a role in multiple intracellular processes, it is not essential to cell survival and PARP1 deletion does not lead to cell death^[24]^. It has been shown that PARP1 deletion leads to partial PARPi resistance, rather than complete because PARP trapping is not the sole mechanism of PARPi action^[24,49]^. Specific point mutations, however, can lead to PARPi resistance as shown with *de novo* resistance to Olaparib in a patient with ovarian cancer^[50]^. Additionally, PARP1 phosphorylation at Tyr907 by tyrosine kinase c-Met increases PARP1 catalytic activity and reduces binding affinity to PARPi leading to PARPi resistance^[51]^.

PARG plays a vital role in the degradation of nuclear PAR, preventing its accumulation and counteracting the effects of PARP1^[26]^. It is now recognized that PARG depletion is associated with PARPi resistance in HR deficient tumours by restoring PARP1 signalling^[26]^.

It remains controversial as to whether overexpression of PARP1 leads to resistance to PARPi. Earlier data suggested that it did lead to resistance resulting in increased PARPi concentrations required to inhibit the enzyme^[52]^. This is in contrast to a retrospective analysis of BRCA1 and PARP1 expression in epithelial ovarian cancer patients that showed no correlation between survival and PARP1 expression^[53]^.

### Suppression of NHEJ

As described earlier, DSBs can be repaired by NHEJ or HR^[24]^. The balance between HR and classical NHEJ (C-NHEJ) is maintained by miR-622, which limits C-NHEJ and promotes HR. Overexpression of miR-622 is associated with decreased expression of genes such as 53BP1, Ku70, and Ku80^[54]^. The Ku complex normally diverts DNA damage response to the C-NHEJ pathway, therefore its decreased expression will lead to activation of HR by the accumulation of MRE11 foci at the sites of DSBs. This rescues the HR deficiency of BRCA1 mutated cells and induces resistance to PARPi^[54]^.

### Replication fork protection

BRCA1 and BRCA2 play a critical role in replication fork protection. BRCA1 promotes end resection of DSBs enabling BRCA2 to load RAD51 onto the exposed DNA strands, thus protecting the replication fork from MRE-mediated degradation^[27]^. Stabilization of the replication fork induces alternate mechanisms of DNA repair resulting in PARPi resistance^[24]^. Pax2 transactivation domain-interacting protein (PTIP) is a DNA damage response protein that is required to recruit MRE11 nuclease for replication fork degradation. Deficiency of PTIP impedes the recruitment of the MRE11 nuclease to stalled replication forks, which in turn protects nascent DNA strands from extensive degradation^[55]^. As a result, there is a reduced level of chromosomal aberrations in BRCA1/2 deficient cells, leading to genomic stability and drug resistance^[55]^.

There is also work demonstrating that EZH2, a histone methyl-transferase, plays a role in replication fork degradation by recruiting nuclease MUS81 to the stalled fork^[56]^. Therefore, loss of expression of EZH2 or MUS81 will lead to PARPi resistance due to the resulting fork stabilization^[56]^. A mi-RNA has been identified, miR-493-5p, that downregulates MRE11, CHD4 and EXO1 in BRCA2 mutant cells and by doing so, preserves replication fork stability^[42]^. It is interesting to note that these mechanisms of resistance leading to replication fork stabilization do not restore HR^[42,55,56]^.

### Drug concentration

Drug concentration of the PARP inhibitor may play a role. The p-glycoproteins (P-gp), also called multi-drug resistance proteins are involved in the efflux of PARPi. Upregulation of ATP-dependent efflux pump *ABCB1* (*MDR1*) gene, which encodes for P-glycoprotein, a multidrug efflux transporter, can lead to resistance to some PARPi due to their enhanced extracellular translocation^[57]^. P-gp inhibitors have been shown to prevent the decrease of PARPi in colon cancer cells^[58]^ and re-sensitize PARPi-resistant BRCA-1 deficient cells to PARPi^[59]^. In the mouse models, PARPi was more effective when P-gp knockout conditions were added to BRCA-1 deficient cells^[60]^. More work is needed in this domain because the resistance to PARPi via upregulation of P-gp has mainly been demonstrated in cell and animal models^[61]^.

### Detecting resistance

Different methods to detect markers of resistance have been explored. Spatial and temporal heterogeneity is a challenge facing personalised medicine in oncology. Ongoing studies are underway exploring the impact of subclonal populations on tumour biology and progression. Serial tumour sampling to monitor clonal evolution poses practical challenges and is currently not standard practice. An alternative approach under investigation is the use of “liquid biopsies”, whereby circulating cell-free tumour DNA (cfDNA) or circulating tumour cells (CTCs) are analysed in the peripheral blood of patients with cancer^[62]^.

The value of cfDNA and serial samples has been investigated in five patients with ovarian cancer with intragenic mutations predicted to restore BRCA1/2 open reading frames, including two patients with multiple independent reversion alleles^[63]^. Reversion mutations were detected only in tumour samples from patients with recurrent disease (5 of 16) and only in cfDNA from three of five patients with a tumour-detected reversion. In this study, findings from a rapid autopsy of one patient with multiple independent reversions demonstrated that reversion-allele frequency in metastatic sites is an important determinant of assay sensitivity. Research continues in this area^[63]^.

It is important this field continues to be explored and practical methods to delineate mechanisms of resistance continue to be tested such as cfDNA if patient outcomes are to improve. In lung cancer, therapeutic advances have continued to be made for patients with EGFR mutations through improved understanding of the mechanisms of resistance to first-generation tyrosine kinase inhibitors such as erlotinib and the subsequent development of agents specific to the identified mechanisms. This may be more challenging due to the large number of potential mechanisms of resistance to PARPi. Ongoing trials such as the NEO study (NCT03548467) are looking to better identify this. This study uses a window of opportunity design to better delineate predictors of response and resistance to PARPi alongside the use of circulating tumour DNA. ARIEL 2, a phase 2 trial investigating rucaparib in relapsed, platinum sensitive high grade ovarian carcinoma, included the collection of biopsies at relapse to also help delineate this^[64]^. Discovering more about this and identifying practical ways to assess this will help improve care for these patients.

### Overcoming resistance

The main cause of resistance appears to be through the restoration of HR. Due to the complexity of this pathway, there are multiple changes beyond the restoration of the BRCA1/2 protein that contribute to this. Addition of agents that disrupt HR such as CDK12, CDK1 and PI3K inhibitors are some of the strategies being investigated to overcome this resistance^[27]^. Furthermore, a clinical trial (NCT03742245) has been designed to assess the synergism between histone deacetylase inhibitors and PARPi in BRCA deficient cells^[65]^. Combination with an ATR inhibitor overcomes PARPi resistance resulting from SLFN11 inactivation and is currently being evaluated in a phase I study (NCT02723864)^[47]^.

There is a host of other clinical trials in progress looking at alternative strategies to maintain sensitivity to PARPi, such as concurrent inhibition of WEE1, VEGFR and mTOR pathways^[66]^. WEE1 is a cell cycle regulator that promotes reversible cell cycle arrest to facilitate DNA repair. WEE1 inhibitors cause the cells to enter S phase with unrepaired DNA and when used in conjunction with PARPi therapy, may mitigate PARPi resistance^[66]^.

Inhibition of angiogenesis with VEGFR inhibitors induces hypoxia in the tumour microenvironment leading to decreased expression of HR repair proteins and therefore enhanced effects of PARPi therapy^[67]^. Phase 3 trials are in progress exploring this strategy in ovarian cancer by combining olaparib and cediranib, a pan-VEGFR inhibitor (NCT02446600 and NCT02502266). In addition, it has been demonstrated that mTOR inhibitors suppress HR repair and synergize with PARPi which may be another approach to preserve PARPi sensitivity^[68]^.

## Conclusion

PARPi have led to improved outcomes in a several tumour types. The importance of HR deficiency is one key factor in predicting patients that are more likely to be sensitive. The likelihood of relapse remains high in these patient populations, and hence defining the mechanisms promoting resistance, and subsequent therapeutic failure remains extremely important. Gaining an understanding of resistance mechanisms may guide combination therapies to overcome resistance. Choosing the best treatment option following the development of resistance to PARPi remains a challenge. Multiple signatures may predict responders and also non-responders. Given the complexity, ongoing research will help better delineate the cohorts that benefit and help define therapies that overcome resistance pathways.

## Declarations

### Copyright

©The Author(s) 2019.
